# Short-term outcomes of a novel salvage procedure for non-reducible patella dislocation post-revision total knee arthroplasty: a 1–4-year follow-up preliminary study

**DOI:** 10.1097/JS9.0000000000002014

**Published:** 2024-09-04

**Authors:** Piotr Dudek, Maciej Kocon, Jacek Kowalczewski

**Affiliations:** Department of Orthopaedics and Rheumoorthopaedics, Centre of Postgraduate Medical Education, Warsaw, Poland

## Introduction

Highlights

- Novel salvage procedure introduced for chronic patella dislocation post-revision total knee arthroplasty (rTKA).
- Surgical technique focused on soft tissue balancing and realignment without extensive muscle mobilization.
- Results suggest effectiveness in correcting patella tracking, stabilizing the extensor mechanism, and regaining knee function.

Patellar instability or dislocation is a significant complication that can arise after revision total knee arthroplasty (rTKA), often resulting in severe functional impairment. The incidence of patellar instability in total knee arthroplasty (TKA) ranges from 1 to 29%^1,2^. Risk factors include improper alignment of prosthetic components, excessive postoperative valgus alignment, prosthetic design geometry, extensor mechanism dysfunction, and anatomical variations like patella alta^3^. Forces generated across the patellofemoral joint during activities can significantly impact the patient’s quality of life post-TKA. Despite extensive documentation on managing chronic patellar dislocations before primary TKA, data on non-reducible patellar dislocation management with well-aligned implants remains scarce^4,5^.

Conservative treatment for patellar dislocation post-TKA is often inadequate, necessitating revision surgery to restore joint function. Corrective procedures for the extensor mechanism include proximal, medial, and distal realignment. Proximal realignment, such as the Insall technique, often needs an extension to distal realignment and lateral release^6^. Medial realignment, including lateral retinaculum release, may require additional procedures like medial structure plication. Distal realignment involves altering the distal extensor mechanism attachment, with tibial tubercle osteotomy (TTO) being effective but risky with poor bone quality or revision implants^7^.

This case series elucidates the challenges and outcomes of non-reducible patellar dislocation post-rTKA. It introduces a novel salvage procedure to address patellar tracking issues, aiming to restore proper alignment and function. The technique is discussed in depth, offering guidance for clinicians managing this complex complication. This salvage procedure is employed in severe cases where standard approaches have not yielded satisfactory results.

## Materials and methods

### Patient selection

This retrospective study included patients who underwent revision total knee arthroplasty (rTKA) at Academic Hospital from 2019 to 2022. Criteria included documented reasons for revision such as implant malposition, patella dislocation, aseptic loosening, or instability. Prosthesis types considered were cruciate-retaining, posterior stabilized, constrained condylar knee, and hinged knee prostheses. A total of 9 patients (5 females, 4 males) aged 45–75 were included, with follow-up ranging from 12 to 45 months. BMI was recorded, and functional status was assessed using the Knee Society Score (KSS) and the Western Ontario and McMaster Universities Osteoarthritis Index (WOMAC) preoperatively and postoperatively (Table 1).

**Table 1:** Patient data and characteristics.

						KSS		WOMAC				
No.	Initials	Age at the time of surgery (years)	BMI (kg/m^2^)	Sex (F/M)	Follow-up (months)	Preoperative	Postoperative	Preoperative	Postoperative	No. previous surgeries	Type of prosthesis “before -> after” revision	Reason for revision surgery
1	G.T.	49	38.4	M	45	33	172	22	86	1	CCK->Hinged	AL., PD.
2	G.R.	45	37.7	M	44	23	89	20	29	2	PS->PS	IM., PD.
3	P.B.	70	36.8	F	31	108	153	71	80	3	PS [NE]	PD.
4	S.B.	75	28.9	M	30	99	165	53	83	2	PS->CCK	IM., PD.
5	M.M.	72	28.4	F	26	11	50	20	29	3	CCK-Hinged	IM., I.,PD.
6	M.L.	73	29.4	F	24	46	114	28	53	2	CR->Hinged	IM., PD.
7	P.B.	65	23.8	F	22	143	175	68	86	1	Hinged [NE]	PD.
8	Z.A.	71	29.4	M	18	150	170	69	85	1	Hinged [NE]	PD.
9	H.K.	71	34.2	F	12	99	146	56	81	2	Hinged->Hinged	IM.,AL.,PD.

AL, aseptic loosening; CCK, constrained condylar knee; CR, cruciate retaining; IM, implant malposiotion; NE, no exchange of prosthesis; PD, patella dislocation; PS, posteriori stabilised.

### Imaging studies

Patients underwent comprehensive imaging, including anteroposterior and lateral X-rays, Merchant view patellar X-rays, and preoperative CT scans to evaluate implant alignment. Postoperative imaging followed the same protocol, with additional CT scans for suspected complications.

### Surgical technique

**Figure 1:**
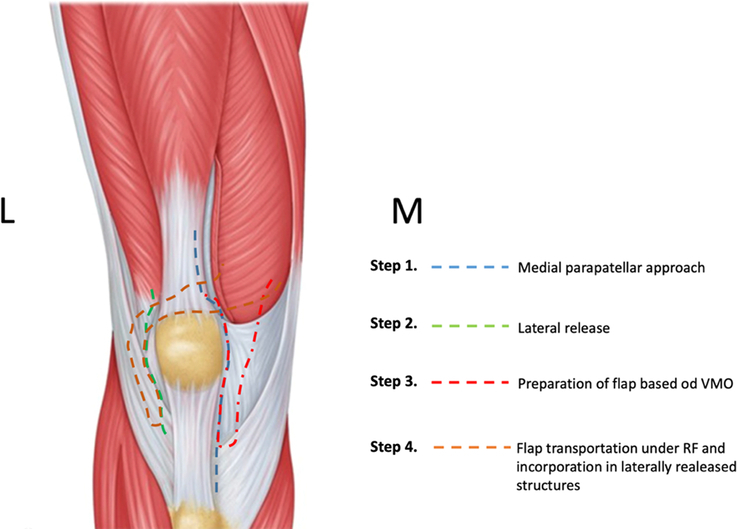
Medial to lateral flap diagram. RF, The rectus femoris muscle. VMO, The vastus medialis oblique muscle.

Patients were positioned according to standard TKA protocol. The existing skin incision was used, and a medial parapatellar approach was performed. Extensive lateral release was done for fixed patellar dislocation. A tissue flap from the medial capsule was transferred under the quadriceps tendon and stabilized with sutures to centralize the extensor apparatus. Closure was meticulously performed. All surgeries were conducted by an experienced arthroplasty surgeon. Written and verbal consent was obtained, and the study complied with the Declaration of Helsinki and was approved by the Ethical Committee (No. 2/2023) (Fig. 1).

### Postoperative rehabilitation

The rehabilitation protocol limited knee flexion to 60° for the first 2 weeks, then 90° for the following 4 weeks, to balance early mobilization with surgical site protection. After 6 weeks, patients resumed full range of motion, aiming for optimal functional recovery.

## Results

The mean KSS increased significantly from 79.11 (SD=52.13) preoperatively to 137.11 (SD=43.70) postoperatively, with a mean improvement of 58 (95% CI 26.57–89.34, *P*<0.05). The Cohen’s d was 1.21. The WOMAC score improved from a mean of 45.22 (SD=22.45) preoperatively to 68 (SD=24.35) postoperatively, with a mean improvement of 22.78 (95% CI 7.48–38.08, *P*<0.05). The Cohen’s d was 0.97. Extension lag reduced from 31° preoperatively to 2° postoperatively. ROM improved from 96.11° (SD=40.91) to 121.67° (SD=22.91), with a mean improvement of 25.56 degrees (95% CI 3.9–47.22, *P*<0.05). The Cohen’s d was 0.77. VAS scores decreased from a mean of 6.67 (SD=0.87) preoperatively to 2.67 (SD=1.58) postoperatively, with a mean reduction of 4 (95% CI 3.17–4.83, *P*<0.001). The Cohen’s d was 3.14.

Two complications were attributed to the surgical technique. Patient no. 2 experienced persistent quadriceps tendon pain that remained unresolved. Patient no. 9 had lateral patella tracking, which required no intervention and maintained a well-functioning extensor mechanism.

The novel surgical technique significantly improved knee function, reduced pain, and enhanced quality of life for patients with persistent patella dislocation post-rTKA. Complications were effectively managed, with satisfactory outcomes. Further research with a larger cohort and longer follow-up is recommended to establish this technique as a standard approach.

## Discussion

Managing patellar dislocation due to soft tissue imbalance following total knee arthroplasty (TKA) and revision total knee arthroplasty (rTKA) with well-positioned implants is a complex challenge requiring meticulous evaluation and intervention. Patellar dislocation significantly affects knee function, causing pain, restricted mobility, and reduced quality of life. Revision surgery with accurate implant positioning is often necessary to restore joint function and stability. Study by Warschawski *et al* ^8^. highlights the importance of correcting implant malposition to manage patella instability post-TKA.

In cases of chronic, non-reducible patellar dislocation, alternative surgical approaches are essential. Realignment procedures targeting the extensor mechanism are crucial for restoring knee function. Matar *et al* ^4^. described a successful extensive proximal extensor mechanism realignment combining lateral release with vastus medialis (VM) muscle advancement. Maintaining VM muscle blood supply is vital for successful outcomes. Proximal realignment suffices for soft tissue imbalance, with distal realignment considered only if proximal measures fail.

Medial patellofemoral ligament (MPFL) reconstruction is also effective for patellar instability post-TKA. Lamotte *et al* ^7^. reported good results with MPFL reconstruction combined with lateral release, although patellar fractures are a serious complication. Grace *et al* ^9^. found proximal realignment sufficient in most cases, with combined realignment needed in fewer cases.

Techniques like tibial tubercle osteotomy (TTO) can be problematic, especially with osteoporotic bone or revision implants requiring tibial stems, increasing nonunion and extensor mechanism rupture risks^10^. The presented salvage technique effectively restores knee function when implant repositioning alone is insufficient.

### Study limitations

The small sample size (9 patients) and retrospective nature without a control group limit generalizability and causal inference. Follow-up periods varied (12–45 months), complicating consistent long-term outcome assessment. Conducted at a single institution, findings may not apply to other settings. Further research with larger, controlled, multi-center studies is needed to confirm these findings.

## Conclusions

Managing patella dislocation after TKA and rTKA is complex and evolving. Successful outcomes require evidence-based practices, innovative surgical techniques, and understanding patella instability mechanisms. Revision surgery is necessary for implant malposition, while proximal, distal, or combined realignment procedures address soft tissue imbalances. This study highlights excellent outcomes from a novel proximal realignment procedure for persistent, non-reducible patella dislocation. Further research, larger studies, and long-term follow-up are needed to refine treatment protocols and optimize surgical interventions.

## Ethical approval

The study was carried out in accordance with the World Medical Association Declaration of Helsinki and was approved by the Ethical Committee of The Centre of Postgraduate Medical Education in Warsaw No. 2/2023.

## Consent

Written informed consent was obtained from the patient for publication of this case report and accompanying images. A copy of the written consent is available for review by the Editor-in-Chief of this journal on request.

## Source of funding

None

## Author contribution

P.D.: conceptualization, data curation, investigation, methodology, writing. M.K.: data curation. J.K.: conceptualization, writing.

## Conflicts of interest disclosure

The authors declare no conflicts of interest.

## Research registration unique identifying number (UIN)

https://www.researchregistry.com/.researchregistry10143. https://www.researchregistry.com/browse-theregistry#home/registrationdetails/66054caf715e5b0028ccf239/.

## Guarantor

Piotr Dudek, Jacek Kowalczewski.

## Data availability statement

The data provided here are accurate to the best of our knowledge. There is no breach of confidentiality.

## Provenance and peer review

Not commissioned; externally peer-reviewed.
